# Outcome of Scapulothoracic Arthroscopy for Painful Snapping Scapula

**DOI:** 10.2174/1874325001711010785

**Published:** 2017-08-21

**Authors:** Saif Ul Islam, Muhammad Naghman Choudhry, Sobia Akbar, Mohammad Waseem

**Affiliations:** Macclesfield District General Hospital, Cheshire, United Kingdom

**Keywords:** Scapulothoracic syndrome, Scapulothoracic arthroscopy, Bursectomy, Snapping, Scapula, Thoracic

## Abstract

**Introduction:**

Patients with scapulothoracic syndrome present with pain in the scapulothoracic area aggravated by overhead and repetitive shoulder movements. The aim of our study was to assess the outcome of scapulothoracic arthroscopic treatment in patients with painful snapping scapula in our institution.

**Methods:**

Fourteen patients underwent scapulothoracic arthroscopic treatment for painful snapping scapula. Pre-operatively, all these patients had a trial of conservative treatment modalities for at least 6 months.

Two portals along the medial border of scapula were used for arthroscopy and instrumentation. In three cases a superior portal was also used. The arm was placed in the “chicken wing” position so that the scapula lifted up from the chest wall. Outcome was assessed using pre and postoperative pain visual analogue score and Oxford Shoulder Score.

**Results:**

Of the fourteen patients included in our study, ten were female and four were male patients. Mean age at the time of surgery was 27.6 years. Mean follow up was 35.7 months. Pain visual analogue score improved significantly from a mean of 8.8 preoperatively to 2.5 postoperatively (P value 0.00002). There was also a significant improvement in Oxford Shoulder Score from a mean of 10.8 to 40.9 (P= 0.00001). Mean crepitus score significantly decreased from 2.6 to 0.21 (p < 0.00001). Crepitus completely resolved in eleven patients. In three there was residual palpable crepitus but they had good pain relief.

**Conclusion:**

Arthroscopic scapulothoracic treatment provides significant pain relief and functional improvement for painful snapping scapula symptoms not responding to non-surgical treatment modalities.

## 1 INTRODUCTION

Snapping scapula or scapulothoracic syndrome occurs due to disruption of the smooth gliding motion between scapula and thoracic cage. It can be chronic and very disabling for patients.

**Fig. (1):**
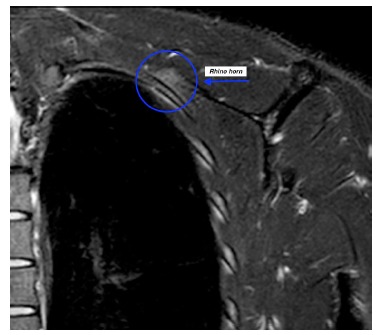
Rhino horn (bony prominence at the superomedial corner of scapula).

**Fig. (2):**
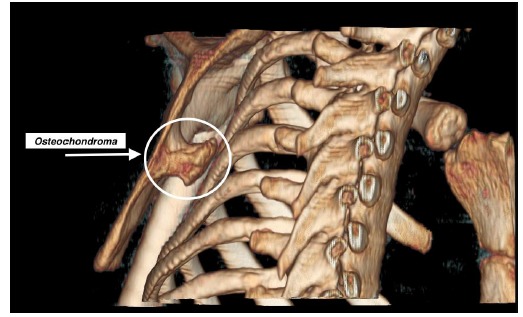
Osteochondroma of Scapula (3-D CT).

**Fig. (3):**
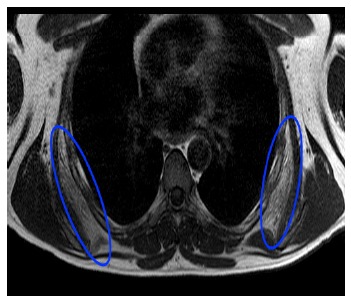
Elastofibroma dorsi.

Boinet first described snapping scapula condition in 1867 [1]. Milch then categorized it into osseous and soft tissue types. Osseous pathology can be due do a variety of bony abnormalities in the scapulothoracic space *i.e.*, Rhino horn (bony prominence at the superomedial corner of scapula) (Fig. **1**), osteochondroma (Fig. **2**), scapula/rib fractures *etc*. Soft tissue pathology is commonly bursitis of any of the 6 bursae around the scapula that include 2 major (supraserratus bursa and infraserratus bursa) and 4 minor bursae (two infraserratus bursae, a supraserratus bursa, and a trapezoid bursa). Bursitis usually develops secondary to dysfunction of scapulothoracic rhythm [2]. It is now well recognised that scapulothoracic syndrome can be secondary to any or a combination of these 3 problems: muscle abnormalities, variations in scapular or thoracic anatomy and bony or soft tissue masses [3] (Fig. **3**).

The patient population is commonly young and active presented with pain in the scapulothoracic area aggravated by overhead and repetitive shoulder movements. During these activities the scapular motion causes audible sound and/or palpable crepitus associated with pain around the scapula [4]. Patients may also describe clicking, crunching, grating or snapping sensation. The severity of this disease has been classified in the literature according to the quality of the audible crepitus. However these classifications are not clear in describing the various grades of palpable and audible crepitus sequentially. Hence, we have proposed a new grading system that, we believe, is clearer and can be used universally in describing the severity of this disease.

**Fig. (4):**
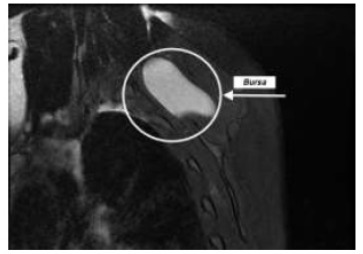
Bursa at superomedial pole of scapula- sagittal proton density fat saturation (PDFS) MRI.

The diagnosis of scapulothoracic syndrome is mainly clinical. Although dynamic ultrasonography can be utilised to identify bursal tissue, MRI scan is most sensitive in identifying small inflammed bursal tissue [5] (Fig. **4**). In some cases CT is useful in identifying any bony incongruity between the anterior aspect of the scapula and chest wall [6].

Non-operative management is the mainstay for this condition. It involves activity modification, nonsteroidal anti-inflammatory drugs and physiotherapy. Corticosteroid and local anaesthetic injections can be a useful aid to the diagnostic workup for scapulothoracic syndrome and can also be therapeutic in some cases.

Acar *et al* have recently described an Extracorporeal shockwave therapy (ESWT) with good outcome results in treatment of scapulothoracic syndrome [7]. Open or arthroscopic scapulothoracic surgical treatment is an option when non-operative modalities have failed. Open bursectomy has been performed with high success rates [8]. However there are limitations to this approach. These include increased initial pain, large incisions with subsequent scarring, a requirement for rhomboid muscle detachment and the need for postoperative immobilization [9, 10].

The arthroscopic treatment for snapping scapula has gained popularity over the last 10 years. So far, limited clinical data have been published describing the results of open or arthroscopic treatment of scapulothoracic bursitis.

The aims of our study are to report the outcome of scapulothoracic arthroscopic treatment in patients with painful snapping scapula in our institution and to introduce a new grading system for this condition.

## 2 METHODS

Fourteen patients underwent scapulothoracic arthroscopic debridement in our institution for painful snapping scapula between June 2009 and August 2015. The duration of their pre-operative symptoms ranged from 1 to 6 years. All of these patients had failed a trial of conservative treatment modalities for at least 6 months. It consisted of activity modification, analgesia and physiotherapy for restoration of normal scapulothoracic kinematics. All patients had a temporary pain relief following a local anaesthetic and steroid injection.

**Fig. (5):**
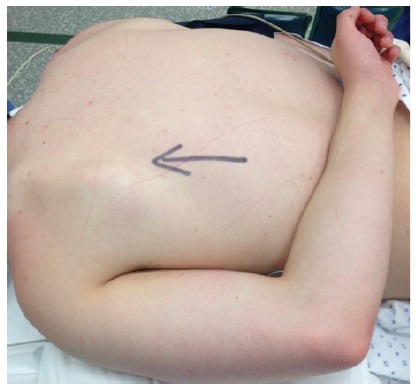
Patient prone with the arm in chicken wing position.

**Fig. (6):**
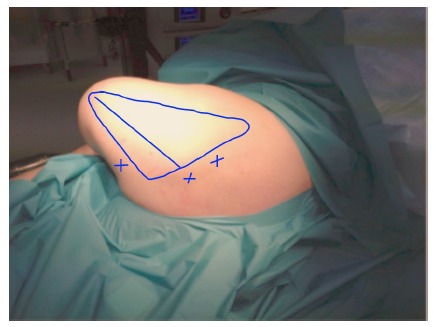
Patient in lateral position and markings for three portals technique.

Operations were performed with the patients in either prone or semi-prone/lateral position. The arm was placed in the “chicken wing” position (arm in full internal rotation with the hand placed on the back), so that the scapula lifted up from the chest wall thus allowing easier access to the scapuluothoracic articulation (Figs. **5** and **6**).

**Fig. (7):**
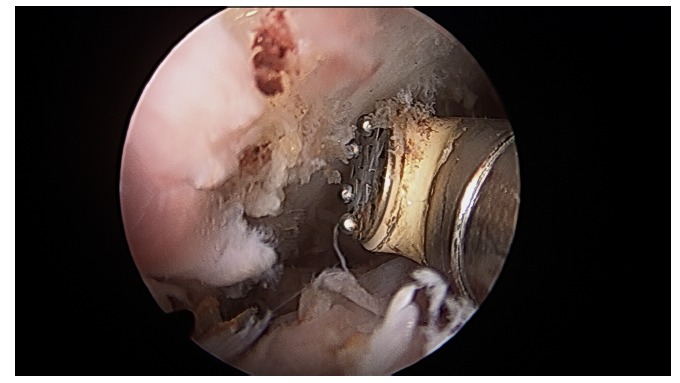
Debridement using radiofrequency device in subscapularis space.

At the start of procedure, 20 ml of normal saline was injected into the scapulothoracic joint to inflate the joint. Two portals along the medial border of scapula were used for arthroscopy and instrumentation in all the cases and an additional third portal was used in three cases to excise the superomedial osseus spur. First portal was the inferior-medial portal placed midway between the spine and the inferior angle of scapula and about 3 cm medial to its medial border. The introducer was passed through the trapezius and rhomboid major to gain access to the serratus anterior space and then through serratus anterior to visualize the suscapularis bursa. A 4.5 mm 30 degrees scope was used for visualization. Fluid pressure was kept at around 50 mm of Hg. Bursa was examined and then the second portal was made 3 cm medial to the spine of scapula using a needle as a guide. Staying 3 cm medial to the medial border of scapula reduces the risk of damage to dorsal scapular nerve and artery and enables a relatively horizontal entry to the bursal spaces thus avoiding accidental entry to the chest cavity. Radiofrequency device and shaver were used for debridement of fibrosis and exuberant bursal tissue in the subscapularis and subserratus bursa (Fig. **7**).

**Fig. (8):**
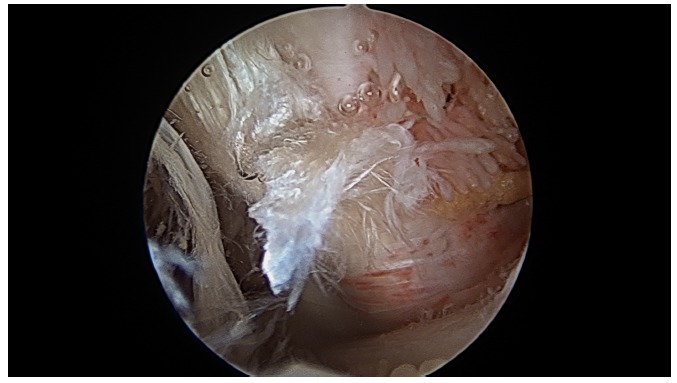
Superomedial bony prominence before resection.

**Fig. (9):**
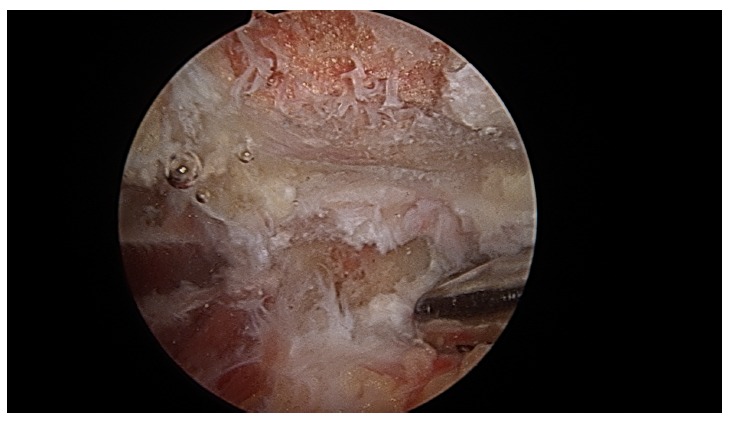
Superomedial bony resection using an arthroscopic burr.

In three cases a superomedial portal was made at the junction of medial third and lateral two third of the superior border of the scapula for excision of prominent osseous spur using an arthroscopic burr (Figs. **8** and **9**).

Outcome was assessed by pre and postoperative pain visual analogue score (VAS) and Oxford Shoulder Score (OSS). Pre and postoperative scores were compared using paired t-test. The significance level was set at P <0.05.

## 3 RESULTS

Of the fourteen patients included in our study, ten were female and four were male patients. Mean age at the time of surgery was 27.6 years (range 16-51 years). The exact nature of surgical intervention was based on the preoperative clinical and MRI/CT findings and the intra-operative arthroscopic findings. Hence, eleven had scapulothoracic bursectomy alone and three had bursectomy and also shaving of superomedial border of scapula. Mean follow up was 35.7 months- range 17 to 46 months (Table **1**).

Visual analogue score (VAS) improved significantly from a mean of 8.8 preoperatively to 2.5 post operatively (P value 0.00008). There was also significant improvement in Oxford Shoulder Score (OSS) from a mean of 10.8 pre-operatively to 40.4 post operatively (P= 0.0007). Based on our new grading system the crepitus score significantly decreased from 2.6 to 0.21 (p < 0.0001). In eleven patients crepitus was completely resolved. In three there was residual palpable crepitus but they had good pain relief. These results are summarized in (Table **2**).

Post operatively all our patients returned to work and the same level of sporting activity except for two patients. One of these is awaiting scapulothoracic arthroscopy on the other shoulder. The other patient is back to work and has returned to playing cricket, however he is able to bat but has not been able to bowl as yet.

## 4 DISCUSSION

Our prospective study of scapulothoracic arthroscopy has shown that in, patients with recalcitrant painful scapulothoracic syndrome who have exhausted 6 months of non-operative modalities, arthroscopic scapula bursectomy gives a good relief of symptoms. All patients were happy with the outcome of their surgery and will recommend this procedure to patients with similar symptoms.

Currently no validated score exists for painful snapping scapula. We used Oxford Shoulder score in our series as it is validated for common shoulder conditions. We also used the validated VAS score for comparison of pre and postoperative pain. A direct comparison of our series to other case series is not possible using Oxford Shoulder Score, as this has not been used in previous studies. However, we can compare our results with previous studies with regards to the VAS score. Harper *et al* reported the results of arthroscopic bony surgery to the superomedial corner of the scapula in 4 patients with painful snapping caused by bony impingement. VAS pain scores reduced from a mean of 8.8 to 2.1 in their series [10]. Similarly, Millett *et al* reported a larger series of patients after scapulothoracic bursectomy and partial scapulectomy. Twenty-one shoulders were evaluated at a mean follow up of 2.5 years postoperatively. Nineteen patients had a bursectomy and scapulectomy while 2 had a bursectomy alone. The American Shoulder and Elbow Surgeons Evaluation Form (ASES) score improved from 53 to 73 points, and VAS pain scores decreased from 9 to 5 [11]. Our results show similar trend to these 2 studies with regard to the VAS pain scores that reduced from a mean of 8.8 to 2.5 in our series.

Merolla *et al* used Western Ontario Rotator Cuff (WORC) score as a post operative outcome measure. They examined 10 patients who underwent arthroscopy for scapulothoracic syndrome. The WORC index significantly improved at 3, 6 and 12 months postoperatively [12]. More recently Menge *et al* published the largest case series of 74 patients who had undergone primary or revision arthroscopic treatment for scapulothoracic syndrome. At a mean follow up of 3.4 years, there was improvement in all postoperative clinical outcomes scores both in primary and revision cases [13]. Their study noted poor outcome scores in patients with lower preoperative mental status score, longer duration of symptoms and older age.

In a critical review of the current evidence about scapulothoracic arthroscopy by Warth **et al*.*, it was concluded that there was only level 4 evidence available. Most of the studies were case reports or case series. Variable outcome measures were used in these studies, such as Constant shoulder score, Western Ontario Rotator Cuff Index, Visual analogue score, *etc*. Hence a meta-analysis or systematic review could not be completed [14].

With regard to the grading systems used for scapulothoracic syndrome, after the initial description of the snapping scapula condition by Boinet, it was subsequently subclassified into 3 categories by Mauclaire in 1904 [15]. These categories were; 1) froissement, a gentle physiologic friction sound; 2) frottement, a louder grating sound that is usually pathologic; and 3) craquement, a consistently pathologic loud snapping sound.

Milch differentiated scapulothoracic crepitus into two categories: a loud, usually painful grating sound caused by a bony lesion, and a less intense sound caused by a soft tissue lesion such as bursitis [16]. Kuhn *et al*, then extrapolated from Milch and proposed that frottement may represent a soft tissue lesion or bursitis, whereas craquement represents an osseous lesion as the source of the painful scapulothoracic crepitus [17]. It is important to appreciate that painful scapulothoracic bursitis may be present without an audible sound. Painless crepitus is more common than painful crepitus and does not usually need to be treated [18].

Accounting for this, we graded the crepitus from 0 to 3. Zero (0) being no crepitus, 1 being palpable but not audible crepitus, 2 being soft audible crepitus and 3 being loud crepitus. This not only involves listening to the quality of crepitus, but also palpating for it. Hence, grade 0 accounts for those patients without any audible or palpable crepitus, where as grade 1 accounts for those painful scapulothoracic bursitis patients without audible sound but a palpable crepitus (Table **3**). Thus our grading system accounts for the whole spectrum of scapulothoraic conditions and can be used as a communication tool among surgeons.

Limitations of our study include a small sample size with relatively short follow up. Nevertheless this study can contribute to the evidence for a multi-center comprehensive study in the future. A multicenter study will be useful in answering questions about the efficacy of the procedure in comparison to non operative treatment modalities and help clarify indications for the procedure [19].

## CONCLUSION

Based on our results we conclude that arthroscopic scapulothoracic treatment provides good pain relief and functional improvement for scapulothoracic syndrome in patients who have failed to respond to nonoperative treatment modalities.

## Figures and Tables

**Table 1:** Patient demographics.

**No**	**Age (years)**	**Gender**	**Symptoms duration (years)**	**Follow-up (months)**
**1**	28	F	2	37
**2**	20	M	6	37
**3**	23	F	5	35
**4**	33	F	5	38
**5**	16	F	2	34
**6**	31	F	5	33
**7**	34	F	2	33
**8**	28	M	1	42
**9**	18	M	3	46
**10**	51	F	2	43
**11**	23	F	4	43
**12**	26	F	3	43
**13**	19	F	1	17
**14**	37	M	4	20

**Table 2:** Pre and Post operative oxford shoulder scores, visual analogue scores and Pre/post operative improvement in crepitus grading. OSS = Oxford Shoulder Scores, VAS = Visual Analogue Scores.

**No.**	**Side operated**	**Osseous Spur Excised**	**OSS**		VAS		Crepitus grade	
			**Pre- op**	**Post op**	Pre- op	Post op	Pre- op	Post op
**1**	Left	No	11	44	9	1	2	0
**2**	Left	No	13	48	10	0	3	0
**3**	Right	No	9	40	7	3	2	0
**4**	Right	No	12	39	9	2	3	1
**5**	Right	No	12	32	8	2	3	0
**6**	Right	Yes	10	40	9	2	2	0
**7**	Left	Yes	9	25	7	4	3	1
**8**	Right	No	8	20	10	6	0	0
**9**	Right	No	13	47	10	1	3	0
**10**	Right	Yes	14	48	8	0	3	0
**11**	Right	No	10	43	10	5	3	0
**12**	Left	No	11	44	10	4	3	0
**13**	Right	No	12	60	9	3	3	0
**14**	Left	No	7	43	8	2	3	1

**Table 3:** New proposed grading of scapulothoracic syndrome.

**Grade**	**Description**
0	No crepitus palpable or audible
1	Crepitus palpable but not audible
2	Soft audible and palpable crepitus
3	A louder grating or snapping sound

## References

[1] Boinet W. (1867). Bulletin de la Societe Imperiale de Chirurgie dr Paris..

[2] Milch H. (1961). Snapping scapula.. Clin. Orthop..

[3] Gaskill T., Millett P.J. (2013). Snapping scapula syndrome: diagnosis and management.. J. Am. Acad. Orthop. Surg..

[4] Pearse E.O., Bruguera J., Massoud S.N., Sforza G., Copeland S.A., Levy O. (2006). Arthroscopic management of the painful snapping scapula.. Arthroscopy.

[5] Ken O., Hatori M., Kokubun S. (2004). The MRI features and treatment of scapulothoracic bursitis: report of four cases.. Ups. J. Med. Sci..

[6] Oizumi N., Suenaga N., Minami A. (2004). Snapping scapula caused by abnormal angulation of the superior angle of the scapula.. J. Shoulder Elbow Surg..

[7] Acar N., Karaarslan A.A., Karakasli A. (2017). The effectiveness of extracorporeal shock wave therapy in snapping scapula.. J. Orthop. Surg. (Hong Kong).

[8] McCluskey G.B. (1990). Surgical management of refractory scapulothoracic bursitis.. Orthop Trans..

[9] Tashjian R.Z., Granger E.K., Barney J.K., Partridge D.R. (2013). Functional Outcomes After Arthroscopic Scapulothoracic Bursectomy and Partial Superomedial Angle Scapulectomy.. Orthop. J. Sports Med..

[10] Harper G.D., McIlroy S., Bayley J.I., Calvert P.T. (1999). Arthroscopic partial resection of the scapula for snapping scapula: A new technique.. J. Shoulder Elbow Surg..

[11] Millett P.J., Gaskill T.R., Horan M.P., van der Meijden O.A. (2012). Technique and outcomes of arthroscopic scapulothoracic bursectomy and partial scapulectomy.. Arthroscopy.

[12] Merolla G., Cerciello S., Paladini P., Porcellini G. (2014). Scapulothoracic arthroscopy for symptomatic snapping scapula: A prospective cohort study with two-year mean follow-up.. Musculoskelet. Surg..

[13] Menge T.J., Horan M.P., Tahal D.S., Mitchell J.J., Katthagen J.C., Millett P.J. (2016). Arthroscopic Treatment of Snapping Scapula Syndrome: Outcomes at Minimum of 2 Years.. Arthroscopy.

[14] Warth R.J., Spiegl U.J., Millett P.J. (2015). Scapulothoracic bursitis and snapping scapula syndrome: A critical review of current evidence.. Am. J. Sports Med..

[15] Mauclaire M. (1904). Craquements sous-capsulaires pathologiques trait’es par l’interposition mus- culaire interscapulo-thoracique.. Bull. Mem. Soc. Chir. Paris.

[16] Milch H. (1950). Partial scapulectomy for snapping of the scapula.. J. Bone Joint Surg. Am..

[17] Kuhn J.E., Plancher K.D., Hawkins R.J. (1998). Symptomatic scapulothoracic crepitus and bursitis.. J. Am. Acad. Orthop. Surg..

[18] Kuhne M., Boniquit N., Ghodadra N., Romeo A.A., Provencher M.T. (2009). The snapping scapula: diagnosis and treatment.. Arthroscopy.

[19] Warme W.J. (2016). CORR Insights(®): Open Surgical Treatment for Snapping Scapula Provides Durable Pain Relief, but so Does Nonsurgical Treatment.. Clin. Orthop. Relat. Res..

